# Visualizing carboxyl-terminal domain of RNA polymerase II recruitment by FET fusion protein condensates with DNA curtains

**DOI:** 10.52601/bpr.2022.210027

**Published:** 2022-04-30

**Authors:** Linyu Zuo, Jiawei Ding, Zhi Qi

**Affiliations:** 1 Center for Quantitative Biology and Peking-Tsinghua Center for Life Sciences, Academy for Advanced Interdisciplinary Studies, Peking University, Beijing 100871, China

**Keywords:** Liquid–liquid phase separation (LLPS), Biomolecular condensates, Single-molecule biophysics, DNA curtains, *In vitro* transcription assay

## Abstract

Many recent references show that living cells can form some membrane-less organelles by liquid–liquid phase separation (LLPS) of biomolecules, like proteins and nucleic acids. LLPS has been confirmed to link with many important biological functions in living cells, and one of the most important functions of biomolecular condensates is in the field of RNA transcription. Many studies confirm that mammalian RNA polymerase II (Pol II) molecules containing the CTD with different phosphorylation level are purposed to shuttle between initiation condensates and elongation condensates of RNA transcription. Traditional ensemble assays often experience difficulties in quantitively and directly recording the transient recruitment of Pol II CTD. Novel single-molecule approach — DNA curtains can be used to directly visualize biomolecular condensates formation and also recruitment of RNA polymerase II (Pol II) carboxyl-terminal domain (CTD) at the target sites in solution and in real time. This method can offer the potential for new insights into the mechanism of gene transcription. Here, we highlight the detailed protocol of DNA curtains method for studying LLPS.

## INTRODUCTION

The membrane acts as a selective boundary to separate different organelles physically in the cell. However, there are also many membrane-less organelles with specific functions, such as P body, stress granule, Cajal body, which are resulted from liquid–liquid phase separation (LLPS) *in vivo* (Anderson* et al.* 2015; Brangwynne *et al*. 2009; Matera 2003; Zhu and Brangwynne 2015). The term LLPS is commonly used in biology referring to the macromolecules in solvent segregated into a concentrated liquid phase from a dilute macromolecules-depleted phase. Since the last century the theoretical hypothesis has been proposed that the biomolecules inside the cell though with a relatively low concentration are able to undergo phase separation in the presence of macromolecular crowding and drive the cytoplasm compartmentalization (Walter and Brooks 1995).

Before LLPS was widely applied to explain biomolecular phenomena, several methods had been used to depict the liquid properties of membrane-less organelles, for example, living cell imaging by time-lapse fluorescence microscopy recorded the fission and fusion events of Cajal bodies (Platani* et al.* 2000), while fluorescence recovery after photobleaching (FRAP) (Reits and Neefjes 2001) and fluorescence correlation spectroscopy (FCS) (Peng* et al.* 2020) techniques help demonstrated the high mobility and exchange dynamics of the proteins inside promyelocytic leukemia protein (PML) nuclear bodies (Weidtkamp-Peters* et al.* 2008), nuclear speckles (Kruhlak* et al.* 2000; Lamond and Spector 2003; Phair and Misteli 2000), and polycomb (Ficz* et al.* 2005). It was not until 2009 that the concept of phase separation was starting to link with biological studies, when the pioneering study described the liquid-like properties and phase separation driven formation of P-bodies *in vivo* (Brangwynne* et al.* 2009), and later the condensates inside the nucleoli (Brangwynne* et al.* 2011). Afterwards, the reconstitution experiments using purified biomolecules have set the paradigm of studying LLPS *in vitro* (Kato* et al.* 2012; Li* et al.* 2012). The early studies have established the definite standard for the LLPS of biomolecules: the roundness of the condensates, the fission and fusion events of the condensates, and the high mobility and exchange dynamics of the molecules inside the condensates measured by FRAP experiment (Boeynaems* et al.* 2018; Mcswiggen* et al.* 2019). These references let us know that these biomolecular condensates formations are mediated by two main factors, which are multivalent interactions among biomolecules and proteins containing intrinsically disordered regions (IDRs) or low-complexity domains (LCDs) (Burke* et al.* 2015; Coletta* et al.* 2010; Li* et al.* 2012; Oldfield and Dunker 2014). Recent studies also indicate that RNA and DNA are crucial players in LLPS (Guo* et al.* 2021; Jain and Vale 2017; Schwartz* et al.* 2013; Zhou* et al.* 2019).

In recent years, a booming number of studies have performed various assays trying to set up the association between the LLPS and biological functions in living cells (Alberti and Dormann 2019; Boija* et al.* 2021). One of the most important functions of biomolecular condensates is that LLPS provides new insights into the working mechanism of the eukaryotic transcription machinery (Boija* et al.* 2018; Cramer 2019; Hnisz* et al.* 2017; Sabari* et al.* 2018).

We first review recent *in vivo* methods to study LLPS and gene transcription (Table 1). First, Tjian and coworkers combined biochemistry assays, like luciferase assay and RT-qPCR and analyses, with different microscopy methods, like confocal fluorescence imaging and live-cell single-particle tracking (SPT), to study the biomolecular condensates of FET fusion proteins and gene transcription (Chong* et al.* 2018). They visualized the EWSLCD hubs formation by integrating an artificially synthetic Lac operator (LacO) array (~50,000 LacO repeats) into the cell genome. They found that EWSLCD hubs can recruit the major subunit of Pol II RPB1, strongly suggesting that FET fusion protein condensates are essential for transcription. Super-resolved imaging also helps visualization of the mediator colocalizing with Pol II and dynamic contacting with gene locus on the chromosome in live cells (Cho* et al.* 2018). Second, the opto-genetic approaches are also strong tools to study LLPS and transcription *in vivo*. Brangwynne and coworkers used a novel optoDroplet system (Wei* et al.* 2020), which can optically control LLPS of IDR by using cryptochrome 2 (Cry2) that can oligomerize under blue light treatment (Shin and Brangwynne 2017). They visualized the forming progress of the FET condensates after artificial nucleation *in vivo*, and these condensates can recruit pol II CTD, activating gene transcription.

**Table 1:** A summary of methods to study LLPS and transcription

Method	RNA transcript detecting technique	Typical application	Reference
*In vivo* methods			
Super resolution imaging	Luciferase report system and RT-qPCR	Visualization of the EWS LCD hubs formation after artificial nucleation by integrating a LacO array, recruitment of Pol II, and gene activation	Chong* et al.* 2018
Super resolution imaging	MS2 endogenous RNA labeling system	Visualization of the mediator condensate dynamics, colocalization with Pol II, and association with gene locus	Cho* et al.* 2018
optoDroplet system	MS2 endogenous RNA labeling system	Visualization of the FET condensates forming progress after artificial nucleation by optical control, recruitment of pol II, and gene activation	Wei* et al.* 2020
*In vitro* methods			
Hydrogel binding assay	Luciferase report system	TAF15 and FUS condensates recruit Pol II CTD	Kwon* et al.* 2013
Reconstituted transcription system	qRT-PCR	Direct observation that the transcription machinery and DNA templates are concentrated into droplets and produce RNA	Henninger* et al.* 2021
DNA curtains	MS2 or UTP-Fluor dye	Direct visualization of FUS fusion protein condensate formation on DNA target site and RNA production	Zuo* et al.* 2021

**Figure 1:**
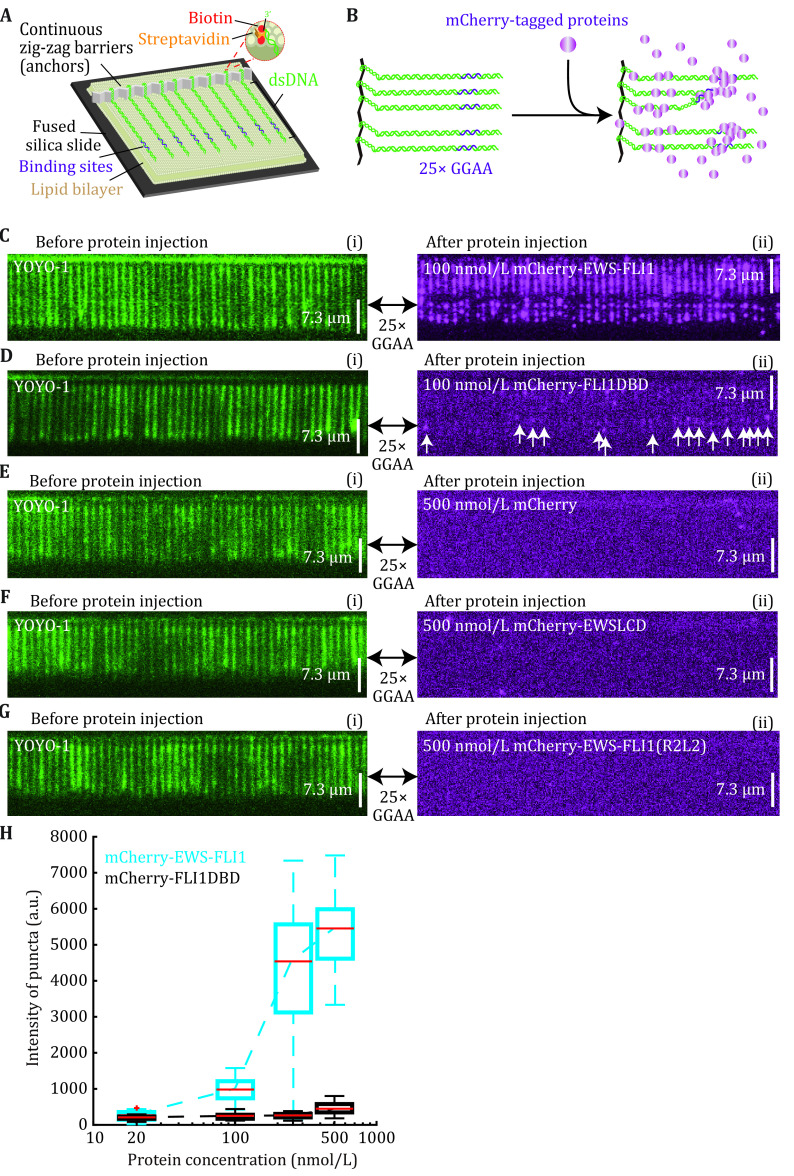
EWS-FLI1 molecules form biomolecular condensates at target sites. **A** Design sketch of DNA Curtains. **B** Schematic of visualizing EWS-FLI1 condensates on DNA Curtains. **C**–**G** Wide-field TIRFM images showing the green signals of YOYO-1 indicating Lambda DNAs with 25× GGAA motifs before protein injection (i) and magenta signals of mCherry-labeled molecules after protein injection (ii). Panels D–G provide control experiments for the mCherry-EWS-FLI1 puncta in Panel C. **H** Boxplot of the puncta intensity at the 25× GGAA target sites of EWS-FLI1 (cyan) and FLI1-DBD (black) versus protein concentration. Adapted with permission from Zuo* et al.* (2021)

**Figure 2:**
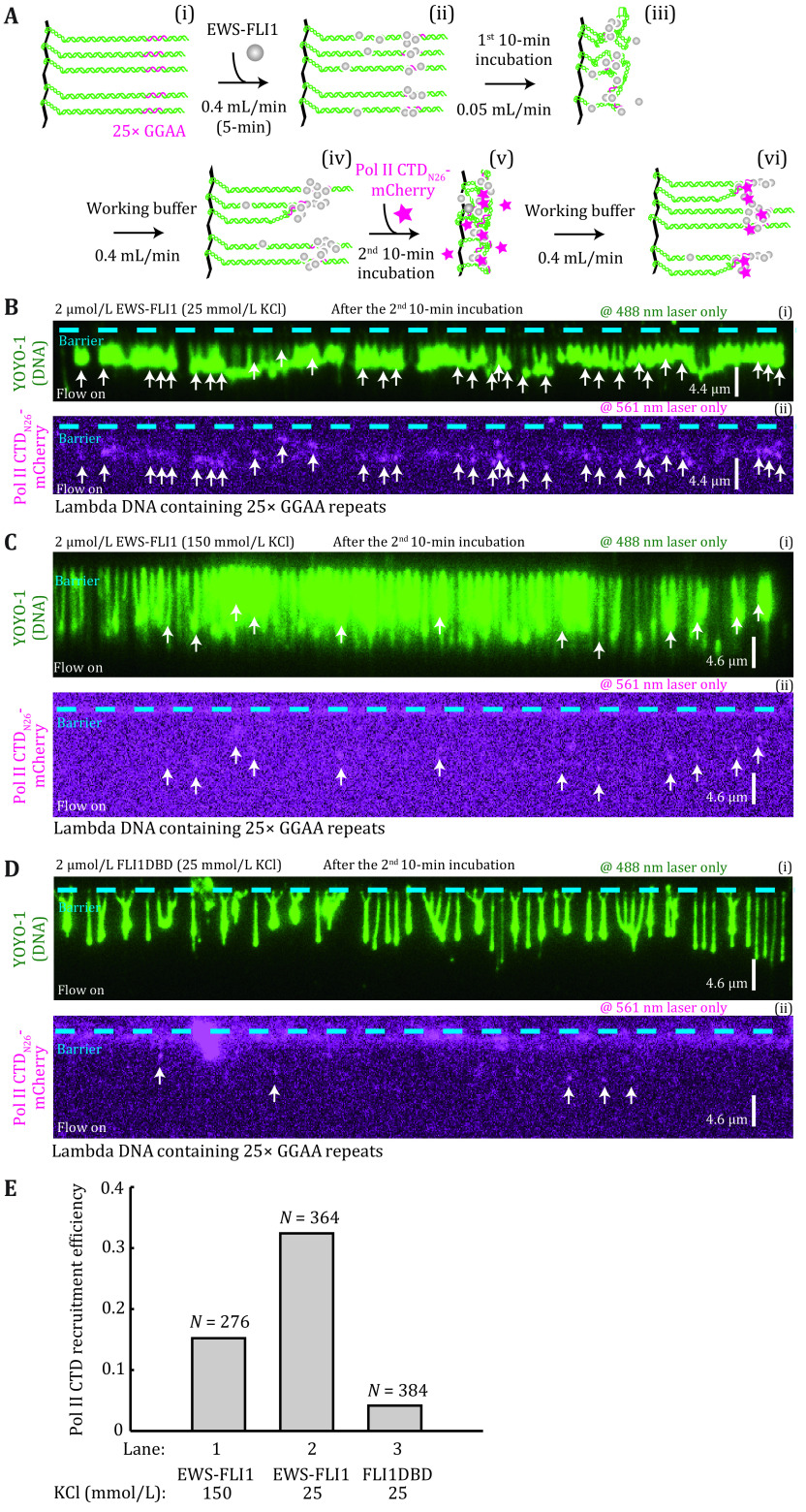


The *in vivo* imaging of the LLPS can demonstrate the dynamic properties of biomolecular condensates and the spatial association of different biomolecules under a physiological state. However, the *in vivo* studies are less convincing in the causality of cellular events. The reconstitution of biomolecular condensates *in vitro* provides direct evidence for the formation and function of phase separated droplets or gels. We next review recent *in vitro* methods to study LLPS and gene transcription (Table 1). First, McKnight and coworkers built up a hydrogel assay to study the molecular interactions in the condensates (Kato* et al.* 2012). They can prepare hydrogels of FET proteins, indicating the biomolecular condensate formation. They observed the hydrogels of TAF15 and FUS can recruit Pol II CTD (Kwon* et al.* 2013). Pol II CTD colocalizing with FUS LCD condensates is demonstrated by Fawzi and his colleagues using nuclear magnetic resonance (NMR) spectroscopy, as a proof of the liquid property of the FUS-Pol II CTD condensate (Burke* et al.* 2015). Second, by mixing reconstituted mammalian transcription system with mediator and other transcriptional activators, Young and coworkers directly observed that the transcription machinery and DNA templates were concentrated into droplets, and also quantified the production of RNA through RT-qPCR (Henninger* et al.* 2021). However, this method has a problem in confirming whether nascent RNA transcripts are actually a consequence of the droplet formation or not. Third, Qi and coworkers (Zuo* et al.* 2021) applied a high-throughput single-molecule technique — DNA curtains (Greene* et al.* 2010; Zhao* et al.* 2017), to directly visualize the condensate formation of the fusion proteins at the target sites (Fig. 1). These condensates can also recruit Pol II CTD at the target sites (Fig. 2), activating gene transcription *in vitro*.

**Figure 2:** EWS-FLI1 condensates can recruit Pol II CTD to the 25× GGAA target sites. **A** Strategy for detecting loci-specific Pol II CTD recruitment by EWSFLI1 condensates. DNA substrate was Lambda DNA containing 25× GGAA binding sites. **B**–**D** Wide field TIRFM images of protein condensates formed on DNA after the 1^st^ round of EWS-FLI1 or FLI1-DBD incubation and 2^nd^ round of Pol II CTD incubation with 488 nm laser on only (i) and 561 nm laser on only (ii), white arrows indicate colocalized EWS-FLI1 puncta and Pol II CTD. **B** 2 µmol/L EWS-FLI1 in 25 mmol/L KCl buffer. **C** 2 µmol/L EWS-FLI1 in 150 mmol/L KCl buffer. **D** Control experiment using 2 µmol/L FLI1-DBD. **E** The Pol II CTD recruitment efficiency, where lanes 1–3 refer to Panels B–D. Adapted with permission from Zuo* et al.* (2021)

In this work, we highlight the detailed protocol of DNA curtains method for studying LLPS. We describe how to use DNA curtains to observe FET fusion protein condensates at DNA target loci, and also how these EWS-FLI1 condensates recruit Pol II CTD to the specific target sites.

## DNA CURTAINS

DNA curtains (Zhao* et al.* 2017), which is a high-throughput single-molecule method, was first developed by Dr. Eric Greene and his research team at Columbia University. This method is designed to visualize protein–nucleic acid interactions *in vitro*, and has already been applied to study many biological questions. For example, the target search mechanism of CRISPR-Cas9 system (Sternberg* et al.* 2014), and the displacement of DNA-binding proteins by DNA helicase (Finkelstein* et al.* 2010). The detailed information about experimental design and typical data and results were explained in the previous review (Greene* et al.* 2010; Qi and Greene 2016; Zhao* et al.* 2017).

## PREPARATION OF DNA CURTAINS

### Materials

#### EWS-FLI1 and Pol II CTD_N26_-mCherry in vitro purification

( 1 ) EWS-FLI1 fused with msfGFP or SNAP tag at N-terminal and CTD_N26_-mCherry were cloned into pRSF-His vector reconstituted from pRSF-Duet plasmid, respectively. Then the plasmid was transfected to *E. Coli* BL21 (DE3) strain and grew on the LB plate overnight.

( 2 ) Pick several single colonies into 5 mL LB medium and grow overnight, then transfer it to 1 L LB medium and shake to cultivate at 37 °C in a 2-L flask until the OD_600_ reaches 0.6–0.8. Add 0.5 mmol/L IPTG into the medium and shake it at 18 °C for 16–20 h. For the expression of CTD, the temperature should be set to 16 °C.

( 3 ) Centrifuge to harvest the cells at 3500 *g* for 25 min and store frozen at −80 °C. 2 L cell pellets were suspended with 60 mL Buffer A containing 50 mmol/L Tris-HCl (pH 7.4), 1 mol/L KCl, 1 mol/L urea, 10 mmol/L imidazole, 1.5 mmol/L β-ME, 5% glycerol, and PMSF protease inhibitor, sonicated for 10 min. Lysates were cleared by centrifugation at 18,000 *g* for 30 min.

( 4 ) Load the supernatant of lysates onto 5 mL pre-equilibrated Ni-NTA resin, later wash the resin with 25 mL Buffer A containing 40 mmol/L imidazole, then elute the proteins with Buffer A containing 500 mmol/L imidazole. Finally, the EWS-FLI1 proteins were stored at −80 °C in Buffer A after further purification by gel filtration with a Superdex 200 column. For CTD purification, Buffer A should be changed to 40 mmol/L Tris-HCl (pH 7.4) and 500 mmol/L NaCl. Protein samples were stored in 500 mmol/L NaCl, at −20 °C as 50% glycerol stock after purification.

#### Liposomes preparation

( 1 ) Clean a 2 mL glass vial with ddH_2_O and ethanol (99%), and then dry it thoroughly in a drying oven.

( 2 ) Rinse a 250 μL glass syringe with chloroform, and then transfer 200 μL lipid master mix (1 g DOPC (1,2-dioleoyl-sn-glycero-3-phosphocholine) (Avanti, Cat. 850375P), 100 mg PEGylated lipids (18:1 PEG2000 DOPE, 1,2-dioleoyl-sn-glycero-3-phosphoethanolamine-N-[methoxy(polyethylene glycol)-2000] (ammonium salt)) (Avanti, Cat. 870273P), and 25 mg biotinylated lipids (18:1 Biotinyl Cap DOPE, 1,2-dioleoyl-sn-glycero-3-phosphoethanolamine-N-(cap biotinyl) (sodium salt)) (Avanti, Cat. 870273P) dissolved in 10 mL chloroform) into the glass vial.

( 3 ) Use nitrogen gas (99% purity) to evaporate the chloroform very gently with continuously rotating the vial in one direction until no liquid is in the vial and put the glass vial in a vacuum drying oven for 16–24 h or even longer.

( 4 ) Add 2 mL fresh lipid buffer (10 mmol/L Tris-HCl (pH 7.5) and 100 mmol/L NaCl) into the glass vial, and incubate it at the room temperature for more than 2 h. Vortex the solution for several times and transfer it to a 5 mL polypropylene culture tube. The solution would be cloudy at this time.

( 5 ) Sonicate the solution on ice with a micro-tip sonicator (750 W, VCX750, SONICS & MATERIALS, INC) to form small unilamellar vesicles. The protocol is: 20% amplitude, 6 s on, 6 s off, 6 cycles, 30% amplitude, 6 s on, 6 s off, 3 cycles, 40% amplitude, 10 s.

( 6 ) Filter the liposomes with a 0.22 μm nylon syringe filter, and then aliquot and store it at 4 °C for one month.

#### Biotinylated Lambda DNA with microsatellite sequence preparation

( 1 ) Microsatellite DNA containing 25× EWS-FLI1 target sites is inserted into Lambda DNA (NEB, Cat. N3013S) XhoI/NheI sites. The ligation product is packaged into MaxPlax™ Lambda Packaging Extracts (Epicentre, Cat. MP5120). After the phage plaque grows bigger, transfer it into LB and co-cultivate with *E. Coli* cells, and harvest the cells by centrifugation, then purify Lambda DNA from the supernatant.

( 2 ) Mix 50 μL Lambda DNA, 1 μL Biotin primer (5’-(Phos)-AGG TCG CCG CCC-BIOTEG-3’) (100 μmol/L), 54 μL ddH_2_O together, and then heat the mix at 65 °C for 5 min and cool down to the room temperature on the bench.

( 3 ) Add 12 μL 10× T4 ligase buffer and 3 μL T4 ligase to the solution, and then incubate the sample at the room temperature for several hours or overnight.

( 4 ) Add 60 μL Buffer A (30% (*m*/*v*) PEG8000 and 30 mmol/L MgCl_2_) to the solution (120 μL), and then rotate the sample at 4 °C for 20–24 h.

( 5 ) Centrifuge the sample at 18,000 *g* for 5 min and then remove the supernatant.

( 6 ) Use 180 μL 70% precooled ethanol to wash the pellet, and repeat the previous step for one time.

( 7 ) Dry the pellet at the room temperature, and then use 100 μL TE150 buffer to dissolve the DNA.

### Methods

In this session, we will introduce DNA curtains in detail. For the accessibility of this technique, we think at this stage it can only be done with collaboration with experts equipped with this technology and instruments. We really hope in the near future, it is possible to set up such instruments by ordinary users easily.

#### DNA curtains flowcell preparation

( 1 ) Prepare a DNA curtains flowcell containing zig-zag nanofabricated barriers, and install the input and output tubes. The detailed protocol was in the previous references (Greene* et al.* 2010; Qi and Greene 2016; Zhao* et al.* 2017).

( 2 ) Use a 3-mL syringe containing 2.5 mL lipid buffer to wash the flowcell, and also carefully check no bubble in the flowcell.

( 3 ) Prepare 1 mL liposome solution (40 μL liposome stock and 960 μL lipid buffer). Inject one third of the solution slowly into the flowcell, and then incubate for 5 min. Repeat this step for another two times.

( 4 ) Wash the flowcell with 2.5 mL lipid buffer slowly, and then incubate at the room temperature for 30 min (“healing”).

( 5 ) Prepare 30 mL BSA buffer (40 mmol/L Tris-HCl (pH 7.5), 2 mmol/L MgCl_2_, 1 mmol/L DTT, and 0.5 mg/mL BSA (Sigma, Cat. A7030)). Wash the flowcell with 2.5 mL BSA buffer from the output direction.

( 6 ) Inject an 800 μL streptavidin buffer (10 μL 1 mg/mL streptavidin (Thermo, Cat. 5888) stock and 790 μL BSA buffer) into the flowcell from the input tubing, and then incubate for 10 min. Repeat this step for another time.

( 7 ) Rinse the flowcell with a 2.5 mL BSA buffer to remove the free streptavidin.

( 8 ) Add 2.5 μL DNA sample containing Biotin labeled Lambda DNA substrates into a 998 μL BSA buffer. Inject one fourth volume of Lambda DNA substrates slowly to the flowcell, and then incubate for 5 min. Repeat this step for another three times.

( 9 ) Turn on the prism typed total internal reflection fluorescence microscopy (TIRFM) (Collins* et al.* 2014; Greene* et al.* 2010; Zhao* et al.* 2017) during the injections in Step 8. Wash the tubing with 10 mL ddH_2_O. Rinse the prism as well as the tubing connector with ddH_2_O, Hellmanex (2%), and ethanol (99%). Prepare an imaging buffer: 40 mmol/L Tris-HCl (pH 7.5), 2 mmol/L MgCl_2_, 1 mmol/L DTT, 150 mmol/L KCl, 0.5 nmol/L YOYO-1 (Invitrogen, Cat. Y3601), and 0.5 mg/mL BSA. Set up the flowcell on the microscope stage and connect it to the microfluidic system.

(10) Flush the DNA sample into the flowcell with a flow rate of 0.03 mL/min for 10 min after the injections in Step 8. DNA can diffuse to the region containing the nanofabricated barriers, which can block and extend DNA in a 10-min incubation. Afterwards, stop the flow for another 30-min incubation. The incubation time is flexible.

#### Inject EWS-FLI1 into the flowcell

( 1 ) Finish all the 10 steps in the last section.

( 2 ) Search and mark the position of the nanofabrication pattern under the bright field.

( 3 ) Stain DNA by YOYO-1 at 0.4 mL/min flow rate for 10 min.

( 4 ) Prepare the EWS-FLI1 solution: dilute mCherry-EWS-FLI1 to 500 nmol/L in 100 μL and inject it into a 50-μL extra loop, and use the blank working buffer to load the sample into the flowcell with 0.4 mL/min flow. Start data acquisition before protein injection, set up the laser power as 20% for 488 nm and 561 nm, and the real laser powers before the prism are 9.9 mW and 16.0 mW. Turn on the laser to start the data acquisition. 100-ms frames are collected at 2-s intervals. Turn off the 488 nm laser after mCherry signals appear in the chamber to avoid signal leaking of YOYO-1.

( 5 ) The mCherry-EWS-FLI1 samples will reach the flowcell after 30 s from the input tubing, and the mCherry signal would cover the first half of Lambda DNA also 2–3 puncta could be seen at the second half of the DNA containing the cloned 25× GGAA binding sites (Fig. 1).

( 6 ) Turn off the flow and incubate for 10 min (Fig. 2A). DNA together with the proteins will shrink back to the barrier and talk with nearby molecules. Turn on the flow at 0.4 mL/min to acquire data in different frames with 2-s intervals.

#### Pol II CTD recruitment on DNA curtains

( 1 ) Repeat the procedure in the last section but replace the mCherry labeled EWS-FLI1 with SNAP-EWS-FLI1. Green puncta could be seen after flushing the SNAP-EWS-FLI1 into the chamber indicated the fusion proteins have already concentrated on DNA.

( 2 ) When 10-min incubation finish, inject 1 μmol/L CTD_N26_-mCherry from 50 μL loop with 1 mL/min flow and stop the flow as the protein flushing into the chamber. Incubate it for 10 min.

( 3 ) Wash out the free CTD_N26_-mCherry with blank buffer at 0.4 mL/min for 3 min, then start data acquisition with 2-s intervals in different frames and switch the flow on and off to check whether the magenta signals of mCherry appear on DNA (Fig. 2). Here the laser power of 488 nm and 561 nm should be set up at 20% and 50% (28.5 mW).

( 4 ) Count the number of magenta puncta that co-localize with green puncta and also can move with DNA, and the total number of extended DNA molecules in the wide-field image in three repeated experiments. We define the proportion of magenta puncta in the total DNA number as Pol II CTD recruitment efficiency (Fig. 2E).

## DATA INTERPRETATION OF DNA CURTAINS

We can use DNA curtains to study FET fusion protein condensates and gene transcription (Zuo* et al.* 2021).

### EWS-FLI1 molecules form biomolecular condensates at target sites

Single EWS-FLI1 molecule can bind to the specific and also non-specific sites of DNA, and a high concentration of EWS-FLI1 can undergo LLPS at target binding loci (Fig. 1). Design sketch of DNA curtains and schematic of visualizing EWS-FLI1 condensates on DNA curtains were shown in Fig. 1A and 1B. The typical data of DNA curtains are wide-field TIRFM images (Fig. 1C–1G). Before protein injection, the TIRFM image of DNA curtains show many parallel green-color lines, and each of these lines represents a Lambda DNA substrate. DNA was stained by YOYO-1, showing the green color. After mCherry-tagged EWS-FLI1 molecules were injected into the flowcell, protein binding information appeared in the wide-field TIRFM image. Interestingly, high-intensity magenta puncta were shown at the 25× GGAA target site (Fig. 1C(ii)). We can also quantitatively measure the puncta intensity as a function of protein concentration (the blue curve in Fig. 1H), and we found that the puncta intensity increased dramatically when EWS-FLI1 concentration increased. As control experiments, FLI1DBD showed a completely different behavior (Fig. 1D and 1H). These data suggested LCD–LCD interactions mainly contribute to the cluster of EWS-FLI1, and the high-intensity puncta in Fig. 1C(ii) are the biomolecular condensates of EWS-FLI1. Only mCherry (Fig. 1E), EWSLCD (Fig. 1F), and EWS-FLI1 mutant R2L2 that cannot bind to DNA (Fig. 1G) were also conducted as control experiments. This kind of DNA curtains experiments can be used to examine biomolecular condensates forming at target sites.

### EWS-FLI1 condensates can recruit Pol II CTD to the 25× GGAA target sites

The strategy for detecting loci-specific Pol II CTD recruitment by EWSFLI1 condensates was shown in Fig. 2A. Here, we designed a two-step DNA curtains experiment. In the 1^st^ step, 2 μmol/L dark EWS-FLI1 was injected into the flowcell and the sample was incubated in the flowcell for 10 min. The aim of this step is to establish EWS-FLI1 condensates at the 25× GGAA target site, like the protocol in the last section. Next, in the 2^nd^ step, we injected 1 μmol/L the N-terminal heptapeptide repeat 1–26 of the human Pol II CTD34 tagged with mCherry (termed Pol II CTD_N26_-mCherry) into the chamber for a 2^nd^ 10-min incubation. Finally, we turned on the flow and performed the data acquisition. For the data acquisition, we first used the 488-nm laser to record YOYO-1 signals, and then turned off the 488-nm laser and turned on the 561-nm laser to record mCherry signals. This protocol can guarantee that no any YOYO-1 signals can leak into the mCherry signal channel, and all mCherry signals come from Pol II CTD_N26_-mCherry. In Fig. 2B(ii), we observed many mCherry signals. Here, DNA curtains method has a great advantage to distinguish whether the fluorescent signals in the flowcell bind to DNA or just randomly stick to the flowcell surface. We can let DNA substrates shrink back by turning off the buffer flow, and those fluorescent signals that can also shrink back with DNA can be confirmed to bind to DNA. In Fig. 2B, we used white arrows to point to puncta of Pol II CTDN26-mCherry that bound to DNA. To confirm these puncta are also colocalized with EWS-FLI1 condensates, we conducted a control experiment by using FLI1DBD (Fig. 2D). As FLI1DBD cannot undergo LLPS at the 25× GGAA repeat, in comparison to the experiment of EWS-FLI1 (Fig. 2B), we only observed few white arrows, proving that the puncta of Pol II CTDN26-mCherry (white arrows) colocalized with EWS-FLI1 condensates in Fig. 2B. By counting the white arrows, we can calculate the Pol II CTD recruitment efficiency in Fig. 2E. We also found the salt concentration can also affect the Pol II CTD recruitment efficiency (Fig. 2C and 2E). This kind of two-step DNA curtains experiments can be used to examine the Pol II CTD recruitment capacity of biomolecular condensates.

## CONCLUSION

The burgeoning diversified fluorescence labeling and imaging methods have been accelerating LLPS studies in the biological field. LLPS has been confirmed to link with many important biological functions in living cells (Alberti and Dormann 2019; Boija* et al.* 2021), and one of the most important functions of biomolecular condensates is in the field of RNA transcription (Boija* et al.* 2018; Cramer 2019; Hnisz* et al.* 2017; Sabari* et al.* 2018). In this work, we review recent experimental *in vivo* and *in vitro* methods for studying LLPS and gene transcription and highlight the detailed protocol of DNA curtains method (Zuo* et al.* 2021). Taken together, the DNA curtains method about LLPS and gene transcription can help us understand the biophysical mechanism of LLPS features, and the new and useful experimental tools mentioned in this work can also be used for cancer therapeutic development in the near future.

## Conflict of interest

Linyu Zuo, Jiawei Ding and Zhi Qi declare that they have no conflict of interest.

## References

[bAlberti2019] (2019). Liquid-liquid phase separation in disease. Annu Rev Genet.

[bAnderson2015] (2015). Stress granules, P-bodies and cancer. Biochim Biophys Acta.

[bBoeynaems2018] (2018). Protein phase separation: a new phase in cell biology. Trends Cell Biol.

[bBoija2018] (2018). Transcription factors activate genes through the phase-separation capacity of their activation domains. Cell.

[bBoija2021] (2021). Biomolecular condensates and cancer. Cancer Cell.

[bBrangwynne2009] (2009). Germline P granules are liquid droplets that localize by controlled dissolution/condensation. Science.

[bBrangwynne2011] (2011). Active liquid-like behavior of nucleoli determines their size and shape in *Xenopus laevis* oocytes. Proc Natl Acad Sci USA.

[bBurke2015] (2015). Residue-by-residue view of *in vitro* FUS granules that bind the C-terminal domain of RNA polymerase II. Mol Cell.

[bCho2018] (2018). Mediator and RNA polymerase II clusters associate in transcription-dependent condensates. Science.

[bChong2018] Chong SS, Dugast-Darzacq C, Liu Z, Dong P, Dailey GM, Cattoglio C, Heckert A, Banala S, Lavis L, Darzacq X, Tjian R (2018) Imaging dynamic and selective low-complexity domain interactions that control gene transcription. Science 361(6400): 361(6400): eaar2555. doi: 10.1126/science.aar2555

[bColetta2010] (2010). Low-complexity regions within protein sequences have position-dependent roles. BMC Syst Biol.

[bCollins2014] (2014). DNA curtains: novel tools for imaging protein-nucleic acid interactions at the single-molecule level. Methods Cell Biol.

[bCramer2019] (2019). Organization and regulation of gene transcription. Nature.

[bFicz2005] (2005). Polycomb group protein complexes exchange rapidly in living *Drosophila*. Development.

[bFinkelstein2010] (2010). Single-molecule imaging reveals mechanisms of protein disruption by a DNA translocase. Nature.

[bGreene2010] (2010). DNA curtains for high-throughput single-molecule optical imaging. Methods Enzymol.

[bGuo2021] (2021). RNA and liquid-liquid phase separation. Non-coding RNA Res.

[bHenninger2021] (2021). RNA-mediated feedback control of transcriptional condensates. Cell.

[bHnisz2017] (2017). A phase separation model for transcriptional control. Cell.

[bJain2017] (2017). RNA phase transitions in repeat expansion disorders. Nature.

[bKato2012] (2012). Cell-free formation of RNA granules: low complexity sequence domains form dynamic fibers within hydrogels. Cell.

[bKruhlak2000] (2000). Reduced mobility of the alternate splicing factor (Asf) through the nucleoplasm and steady state speckle compartments. J Cell Biol.

[bKwon2013] (2013). Phosphorylation-regulated binding of RNA polymerase II to fibrous polymers of low-complexity domains. Cell.

[bLamond2003] (2003). Nuclear speckles: a model for nuclear organelles. Nat Rev Mol Cell Biol.

[bLi2012] (2012). Phase transitions in the assembly of multivalent signalling proteins. Nature.

[bMatera2003] (2003). Cajal bodies. Current Biology.

[bMcswiggen2019] Mcswiggen DT, Mir M, Darzacq X, Tjian R (2019) Evaluating phase separation in live cells: diagnosis, caveats, and functional consequences. Genes Dev33(23-24): 1619-1634

[bOldfield2014] Oldfield CJ, Dunker AK (2014) Intrinsically disordered proteins and intrinsically disordered protein regions. Annu Rev Biochem, 83: 553-584

[bPeng2020] (2020). Phase separation at the nanoscale quantified by dcFCCS. Proc Natl Acad Sci USA.

[bPhair2000] (2000). High mobility of proteins in the mammalian cell nucleus. Nature.

[bPlatani2000] (2000). *In vivo* analysis of Cajal body movement, separation, and joining in live human cells. J Cell Biol.

[bQi2016] (2016). Visualizing recombination intermediates with single-stranded DNA curtains. Methods.

[bReits2001] (2001). From fixed to FRAP: measuring protein mobility and activity in living cells. Nat Cell Biol.

[bSabari2018] (2018). Coactivator condensation at super-enhancers links phase separation and gene control. Science.

[bSchwartz2013] (2013). RNA seeds higher-order assembly of FUS protein. Cell Rep.

[bShin2017] (2017). Liquid phase condensation in cell physiology and disease. Science.

[bSternberg2014] (2014). DNA interrogation by the CRISPR RNA-guided endonuclease Cas9. Nature.

[bWalter1995] (1995). Phase separation in cytoplasm, due to macromolecular crowding, is the basis for microcompartmentation. FEBS Lett.

[bWei2020] (2020). Nucleated transcriptional condensates amplify gene expression. Nat Cell Biol.

[bWeidtkampPeters2008] (2008). Dynamics of component exchange at PML nuclear bodies. J Cell Sci.

[bZhao2017] (2017). Visualizing biological reaction intermediates with DNA curtains. J Phys D: Appl Phys.

[bZhou2019] (2019). Mechanism of DNA-induced phase separation for transcriptional repressor VRN1. Angew Chem Int Ed Engl.

[bZhu2015] (2015). Nuclear bodies: the emerging biophysics of nucleoplasmic phases. Curr Opin Cell Biol.

[bZuo2021] (2021). Loci-specific phase separation of FET fusion oncoproteins promotes gene transcription. Nat Commun.

